# Coordination of Genomic RNA Packaging with Viral Assembly in HIV-1

**DOI:** 10.3390/v8070192

**Published:** 2016-07-14

**Authors:** Chris Hellmund, Andrew M. L. Lever

**Affiliations:** Department of Medicine, University of Cambridge, Cambridge CB2 0QQ, UK; ch544@cam.ac.uk

**Keywords:** HIV-1, RNA, packaging, assembly

## Abstract

The tremendous progress made in unraveling the complexities of human immunodeficiency virus (HIV) replication has resulted in a library of drugs to target key aspects of the replication cycle of the virus. Yet, despite this accumulated wealth of knowledge, we still have much to learn about certain viral processes. One of these is virus assembly, where the viral genome and proteins come together to form infectious progeny. Here we review this topic from the perspective of how the route to production of an infectious virion is orchestrated by the viral genome, and we compare and contrast aspects of the assembly mechanisms employed by HIV-1 with those of other RNA viruses.

## 1. Introduction

Production of infectious viral progeny is the ultimate aim of the period of residence of a virus within a cell. Formation of viable virion particles during the later stages of the replication cycle is complex and requires coordination, by the virus, of a large number of cellular and viral factors many of which are proteins. Often overlooked in RNA viruses is the central controlling role of the one molecule that is present throughout this process—the viral genome. The packaged RNA is a key player in this highly organized sequence of events and they illustrate the protean capabilities of this molecule in directing cellular functions.

## 2. Overview 

**Figure 1:**
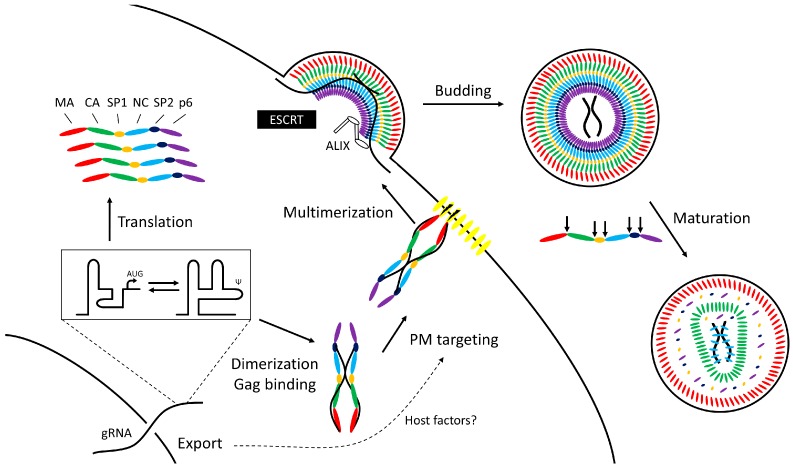
Overview of the assembly pathway of human immunodeficiency virus type 1 (HIV-1). The viral protein Rev facilitates efficient export of unspliced genomic RNA (gRNA) from the nucleus. Upon entering the cytoplasm, the gRNA can adopt two conformations, which permit either translation or dimerization to form a structure which is selectively packaged by group-specific antigen (Gag). Gag trafficking to the plasma membrane is linked to the mechanism of nuclear export of gRNA, and requires RNA binding to the matrix domain to inhibit binding to intracellular membranes. Multimerized Gag recruits the host endosomal sorting complexes required for transport (ESCRT) machinery for budding, including ALIX, which binds both the p6 and nucleocapsid (NC) domains. Following release immature virions undergo proteolytic maturation (cleavage sites in Gag indicated by arrows) leading to production of infectious virus particles.

Following the export of unspliced human immunodeficiency virus type 1 (HIV-1) mRNA from the nucleus into the cytoplasm, the 9 kb transcript plays a central role in the formation of new infectious virions (Figure 1). It serves as the template for translation of the major structural protein group-specific antigen (Gag) and, through ribosomal frameshifting [1], Gag-Pol, encoding the viral protease, reverse transcriptase, and integrase enzymes. Through specific interactions using *cis* acting elements (packaging signals—Ψ), the unspliced RNA is also packaged into virions as the viral genomic RNA (gRNA). Dimerization of gRNA allows recognition and capture by the Gag protein. gRNA, however, is not a passive player in HIV-1 virus production; indeed, RNA-directed control of assembly is evident in other retroviruses, but also in more distantly related RNA viruses.

This review traces the journey taken through the cell by RNA genomes, leading to viral assembly and release, highlighting the experimental data that demonstrates a role for the RNA in late stages of the virus replication cycle. Although focusing on HIV-1, we refer to other RNA viruses to illustrate themes common to different viral families. Critical steps leading to RNA encapsidation regulated by the genome can be briefly summarized thusly:

- ***Translation:*** In HIV-1 and feline immunodeficiency virus (FIV) gRNA control begins at the stage of translation to produce Gag and Gag-Pol. A conformational RNA switch in the 5’ untranslated region (UTR) of the RNA affects the balance between translation and packaging from the same template by altering exposure of the start codon and the elements required for genome dimerization and packaging [2,3,4,5,6]. Moloney murine leukemia virus (MoMuLV) similarly uses an RNA structural change to expose Gag binding sites upon dimerization [7,8].
- ***Genome capture:*** Viral RNA molecules act as scaffolds tethering adjacent Gag proteins through their nucleocapsid (NC) domains [9,10] allowing the newly-transcribed HIV-1 Gag to form oligomers in the cytoplasm [10,11] before trafficking to the plasma membrane where there is evidence that targeting to membrane lipids is linked to the process of gRNA nuclear export [12,13,14].
- ***Virion particle formation:*** Multimerization of Gag to form immature virus particles occurs at the plasma membrane; HIV-1 gRNA act as nucleation sites for the assembly of immature virions [15,16,17]. Alternative models exist for RNA viruses that transport their gRNA into a preformed capsid. A prototypic example is *φ*6 [18,19]. Despite differences in the mechanism of packaging, gRNA coordinates the process in both.
- ***Release from the cell:*** HIV-1 Gag engages with the host endosomal sorting complexes required for transport (ESCRT) machinery to bud from the cell [20,21]; subsequent morphological maturation of the virion occurs by proteolysis of Gag by the viral protease. These linked processes can be disturbed by mutations in the *cis*-acting packaging elements [22,23,24,25,26] suggesting that gRNA binding to Gag is important for virus maturation. The NC domain of Gag serves as a docking site for the ESCRT proteins TSG101 and ALIX [26,27] in addition to their better-studied binding sites in the p6 domain. In the case of ALIX, at least, RNA and membrane lipids appear necessary to stabilize the interaction with Gag [28].

## 3. RNA Structural Switches Affecting Translation and Packaging

Packaging of the HIV-1 genome is a highly specific process. The major structural protein Gag packages dimers of full-length viral RNA with much greater specificity than spliced viral and cellular RNA species, due to the presence of highly conserved *cis*- and *trans*-acting packaging determinants [29,30]. The core *cis*-acting sequences involved in packaging include stem-loop 3 (SL3) [31] and stem-loop 1 (SL1) [32] in the 5’ UTR, but sequences in the Gag coding region and elsewhere in the genome also appear to contribute [32,33,34]. Sequences and structures implicated in packaging span across the major splice donor, facilitating preferential incorporation of unspliced RNA over spliced RNA species (which encode other essential genes but are insufficient to act as a genome if packaged). Recent nuclear magnetic resonance (NMR) data has suggested that the splice donor anneals with upstream sequences to produce a three-way junction structure which enables packaging [35] in contrast to data derived from other techniques, including single-molecule fluorescence resonance energy transfer (smFRET) [36] and small-angle X-ray scattering (SAXS) [37], in which the splice donor is seen to adopt the traditional hairpin stem-loop. The intrinsic flexibility of RNA and its ability to adopt alternative conformations under different experimental conditions undoubtedly contribute to the diversity of predicted structures for the HIV-1 leader region. In contrast, the HIV-2 packaging signal is present upstream of the splice donor [38], thus packaging specificity is achieved by newly-produced Gag interacting with the mRNA from which it is translated [39]. A domain including two zinc finger motifs found in the NC region of Gag serves to mediate specific recognition of the packaging signal in the 5’ UTR [40]. The zinc fingers are flanked by basic residues that are important for non-specific interaction with RNA [41].

Unspliced gRNA acts as both a template for translation and as the genome to be packaged, implying a requirement for a control mechanism to regulate the balance between these mutually exclusive processes. There is long established evidence for the HIV-1 leader RNA adopting different conformations from studies using differences in electrophoretic mobility [42]. Advances in RNA structural probing have shown these different conformations are a result of a structural switch, which regulates the processes of translation and packaging [2,3,4,5]. In monomeric gRNA, the dimerization initiation site (DIS) at the tip of SL1 is occluded by intra-strand base pairing with a motif in the U5 region, whilst the Gag start codon is accessible for translation. The structural rearrangement involves exposure of SL1 and SL3 to enable genome dimerization and occlusion of the start codon by base pairing with the U5 motif [4,5]. The dimer structure produced following this switch appears to be the structure recognized by Gag for packaging to occur [15,17,43].

In vitro, the structural change in the 5’ UTR can be triggered by binding of NC protein [2]. During infection, isolated NC protein would not be available at this stage since proteolytic cleavage of Gag does not commence until virus budding has started. Nevertheless, this is consistent with the observation that at low concentrations Gag stimulates translation, whilst at a higher concentration it is inhibitory [44]. This is analogous to a mechanism adopted by bacteriophage MS2, where coat protein binds to the translational operator of the replicase gene to block translation [45]. MS2 also regulates translation of its maturation protein through delayed folding of an RNA cloverleaf, which conceals the Shine-Dalgarno sequence necessary for ribosome binding, allowing a burst of translation before folding is complete [46]. There is also evidence showing that binding of tRNA^Lys3^, the host RNA used to prime HIV-1 reverse transcription, promotes formation of the dimerization-competent structure over the translation-competent structure [47,48].

Although distinct from HIV-1 in overall structure, the packaging signal region of another lentivirus, FIV, also adopts two conformations that regulate the balance between translation, dimerization and packaging [6]. The more distantly related gammaretrovirus, MoMuLV, also utilizes a structural switch that exposes high affinity NC binding sites when gRNA dimerizes [7,8]. It is not surprising that many examples of RNA structural switches exist in RNA viruses, since these mechanisms enable an additional level of control of virus replication without expanding the genome size. Exploiting RNA structures to regulate mutually exclusive processes is not limited to retroviruses. Positive-sense single-stranded RNA viruses use their genome as both a template for translation (occurring in the 5’-3’ direction), and minus-strand synthesis (occurring in the 3’-5’ direction). A cloverleaf structure in the 5’ UTR of poliovirus regulates this balance through enabling the competing binding of a cellular and a viral protein [49]. Turnip crinkle virus (TCV) uses a structural switch to different effect. In order to reduce the build-up of deleterious mutations in progeny, newly-synthesized RNA harbors an RNA structure unfavorable for RNA-dependent RNA polymerase (RdRp) binding, favoring replication from the infecting parental RNA species (“stamping machine replication”) [50].

## 4. Spatiotemporal Dynamics of the Interaction between Group-specific Antigen (Gag) and Genomic RNA (gRNA)

In recent years a clearer picture has emerged of the spatiotemporal dynamics of the HIV-1 Gag-gRNA interaction, thanks to elegant studies which enable visualization of the two molecules simultaneously in live cells [16,51,52,53,54] and isolation of Gag-gRNA complexes from different cellular compartments [34,55]. Immunoprecipitation of Gag with subsequent quantitative reverse transcription PCR (qRT-PCR) of bound RNA showed that gRNA can be detected when Gag is pulled down from the cytoplasmic fraction of infected cells [55], suggesting that the initial interaction of Gag with gRNA takes place in the cytoplasm.

Recent studies using RNA constructs containing tags for binding of fluorescent fusion proteins suggested that gRNA moves through the cytoplasm in a random diffusive manner, both in the absence and presence of Gag [56], implying that Gag is not required for RNA transport to the plasma membrane. Signals indicating the presence of Gag did not appear until the RNA reached the plasma membrane, however oligomers of Gag containing less than around 20 copies cannot be detected with this method [16]. Direct imaging studies suggest that the number of Gag molecules trafficking the gRNA to the plasma membrane may be below this detection threshold [51]. In agreement with immunoprecipitation findings [55], FRET data showed that oligomerization of Gag begins in the cytoplasm, and is dependent on its NC domain and in particular the second zinc finger motif [11]. A similar observation was made in Rous sarcoma virus (RSV) where the NC domain was demonstrated to be essential for cytoplasmic oligomerization [10]. The assembly of oligomers through NC does not require specific interactions with viral RNA, since replacement of the domain with dimerizing leucine zipper motif restores the assembly function [9], however following capture of gRNA it is likely that the viral genome is involved.

In summary, upon entering the cytoplasm following transcription and nuclear export, HIV-1 gRNA acts initially as a template for Gag translation, but following a structural switch in the 5’ UTR [2,3,4,5] high affinity Gag binding sites are exposed. This switch is likely mediated by binding of the NC domain of Gag to gRNA [2]. The non-specific RNA binding properties of NC then enable apposition of NC domains from adjacent Gag molecules to form oligomers [51,55], which traffic to the plasma membrane where higher order multimerization of Gag can begin [55].

## 5. Targeting Gag to the Plasma Membrane

The matrix (MA) domain of HIV-1 Gag undergoes co-translational addition of myristic acid to its N-terminal glycine, ultimately enabling it to embed into the plasma membrane for early virion assembly [57]. Initial sequestration of myristic acid in a hydrophobic pocket in MA is reversed upon binding of the hydrophobic pocket to PI(4,5)P_2_ (a phospholipid found exclusively on the plasma membrane), enabling a stable anchor to form specifically at this site and avoiding unproductive anchoring to internal membranes [58,59].

Apart from the NC region, the MA domain of HIV-1 Gag can also bind nucleic acid [34,60,61,62,63,64], including viral gRNA [60,65,66]; MA domains of bovine leukemia virus (BLV) [67] and RSV [68] have similar properties. The membrane and nucleic acid binding functions of MA overlap, since the interaction between MA and fluorescently labelled HIV-1 RNA is abolished when the two molecules are incubated in the presence of liposomes containing PI(4,5)P_2_, but not with control liposomes lacking this phospholipid [64]. These results suggest that RNA and plasma membrane lipids compete for binding.

Mutation of lysine residues at MA positions 25 and 26 to threonine leads to enhanced binding to liposomes lacking PI(4,5)P_2_, demonstrating that certain residues in MA are responsible for inhibiting promiscuous binding of the protein to internal cellular membranes [69]. Since these residues also bind to RNA, it was hypothesized that RNA binding inhibits the interaction of MA with internal membranes. Indeed, RNase treatment of WT Gag prior to incubation with liposomes that lack PI(4,5)P_2_ causes significantly more Gag to bind to the liposomes but, in the presence of the K25T/K26T mutations, this increase in binding is reduced.

An RNA aptamer, identified by in vitro genetic selection [61], with a high degree of homology to a region of HIV-1 gRNA, was shown in a later study to bind MA in the same region which binds PI(4,5)P_2_ [70], suggesting that gRNA may act as a negative regulator to ensure that Gag binds specifically to the plasma membrane.

The domains of Gag are connected by flexible linkers and in solution MA appears able to fold back into close proximity to NC [71]. Addition of RNA to purified Gag causes it to condense [72]; thus, it is plausible that the gRNA bound at NC can interact with residues in MA. However, results from cross-linking-immunoprecipitation (CLIP) sequencing from infected cells reveal that following removal of MA from the rest of the Gag precursor, tRNAs are the major RNA species that associate with MA [34].

Defects in Gag localization to the plasma membrane are linked to impaired export of HIV-1 gRNA from the nucleus [13,14,73]. Although HIV particle assembly is notoriously inefficient in murine cells [74] replacing the Rev-responsive element (RRE) with a tetramer of the constitutive transport element (CTE) from Mason-Pfizer monkey virus (thus altering the mechanism of nuclear egress) greatly enhances Gag trafficking to the plasma membrane and subsequent virion assembly [12]. Defective membrane targeting of Gag derived from RRE-containing transcripts can be overcome by mutations in MA that control the myristyl switch mechanism [13]. Replacement of the RRE of HIV with the post-transcriptional regulatory element (PRE) of hepatitis B virus disrupts Gag assembly at the plasma membrane in human cells [14]. This can be rescued by replacing MA with other membrane-targeting motifs or by co-expression with RRE-containing gRNA, provided that the MA domain is not mutated. These data suggest that it is not the presence of gRNA, per se, but its pathway through the nuclear envelope to the cytoplasm that influences the ability of MA to target to the plasma membrane. The mechanism behind these observations is not clear. One proposal is that helicases interacting with Rev in the nucleus have effects on gRNA that alter its function in the cytoplasm [75].

## 6. Assembly of Virions and Packaging

Once at the plasma membrane, oligomers of Gag begin to assemble into higher-order structures mediated by contacts between adjacent MA, CA, and NC regions [11]. This is accompanied by dramatic changes in the RNA motifs that are recognized by Gag [34], likely due to a change in arrangement of NC within the higher order structure. Gag switches from a preference for packaging signal sequences in the 5’ UTR to more general binding to A-rich motifs throughout the viral genome, enabling the genome to act as a scaffold upon which multiple copies of Gag can bind for formation of immature virions. The importance of RNA in MoMuLV particle stability was demonstrated by deletion of the packaging signal whereupon virions switched to packaging cellular RNAs, indicating that RNA was required for assembly. RNase treatment of virions caused them to degrade showing that RNA provides a framework which stabilizes Gag assembly [76]. Similarly, in HIV-1, mutation of RNA-binding residues in NC causes virions to degrade soon after being released from the cell [77]. NC mutant virions also contain abnormally high levels of prematurely reverse-transcribed viral DNA—a phenotype also observed with budding-defective virions—highlighting a link between packaging, budding, and proteolytic processing [78,79].

HIV-1, like other retroviruses, packages two copies of its genome, detected as a stable non-covalently-linked dimer in virions. Whether Gag recognizes a dimer or two monomers that subsequently dimerize has been the subject of lengthy debate (reviewed in [80]) complicated by the fact that the dimerization and packaging sequences in the 5’ UTR overlap. However more recent imaging and biochemical studies suggest that dimerization occurs before packaging, with dimerization being observed both in the cytoplasm and at the plasma membrane [15,16,81]. The ability of gRNAs to dimerize in the cell is dependent on the presence of Gag (likely acting as a chaperone) and an intact SL1 [81]. The latter finding is consistent with the observation that recombination between subtypes with discordant DIS sequences is rare [43]. Studies in which the DIS is duplicated in a single gRNA clearly show encapsidation of monomers [15,17] suggesting that packaging is initiated following recognition of an RNA structure unique to the dimer.

In other RNA viruses, such as MS2 and satellite tobacco necrosis virus (STNV), multiple packaging signals are spaced in such a way as to allow cooperative binding of coat protein subunits [82,83,84]. In these viruses, viral gRNA acts as a template for capsid multimerization, guiding the coat protein in place to assemble the capsid.

In the above examples RNA acts as a nucleator for assembly of the viral capsid. An alternative, but less common, method by which viruses package their genomes is to produce the capsid and then for gRNA to be actively transported into the preformed structure, in a manner similar to some DNA viruses, such as the herpesviruses. A well-studied example of this mechanism in RNA viruses is the bacteriophage *φ*6, a double-stranded RNA virus which packages exactly one of each of three genome segments into its capsid [19]. The packaging of the three segments, small (S), medium (M), and large (L), is highly coordinated—packaging of the M segment requires that the S segment is already packaged and, likewise, the L segment cannot be packaged before the M segment [18]. To explain this phenomenon a model has been proposed whereby the empty virion contains binding sites specific to unique sequences in the S segment. Packaging of the S segment causes the capsid to expand to reveal M segment binding sites, and so on [19]. This model is supported by cryo-EM studies that show the capsid is capable of undergoing a dramatic structural reorganization during expansion [85,86]. Altering the size of the genome causes the virus to compensate by packaging more or fewer molecules of RNA [87], consistent with the idea that an optimal amount of RNA needs to enter the capsid to fill it and trigger the next round of packaging. This contrasts with HIV-1 which uses copy number recognition (i.e., two gRNAs will be packaged almost regardless of size) [15].

Packaging in *φ*6 thus illustrates an alternative to the ‘nucleation’ mechanism of assembly in HIV‑1 and other RNA viruses. However, the common theme is that gRNA actively influences packaging and infectious particle assembly.

## 7. Viral Budding

HIV-1 hijacks the host ESCRT machinery to bud from the plasma membrane, recruiting TSG101 and ALIX through the PTAP and YPXnL motifs, respectively, both of which are found in the p6 domain of Gag [20,21]. ESCRT is composed of four complexes of proteins (termed ESCRT-0–ESCRT-III) which are normally involved in the formation of cellular multivesicular bodies and in cytokinesis (membrane fission events that are topologically equivalent to enveloped virus budding). Their relatively recent discovery means that additional functions are still being identified [88].

The PTAP motif in p6 recruits TSG101 (a member of ESCRT-I) which signals through ESCRT‑II [89]—which also appears to be involved in gRNA trafficking [90]—to ESCRT-III. An alternative pathway for ESCRT-III activation is via ALIX (which interacts with p6 via the YPXnL motif) binding directly to ESCRT-III components. ESCRT-III subunits form long spirals that constrict the budding neck to facilitate scission.

In addition to the PTAP and YPXnL motifs in p6, in vitro pulldown experiments have shown that ALIX and TSG101 also interact with the NC domain of Gag [26,27]. This raises the possibility that the packaged RNA, which binds to NC, is involved in recognition of Gag by the host ESCRT proteins. Conflicting reports existed on the requirement for RNA in the interaction between ALIX and NC [26,91], however it was shown that the ALIX residues involved in the interaction, like those at the NC interface, are positively-charged [91], suggesting that the negatively-charged RNA may act as a ‘bridge’ between the two proteins. A more recent study clarified the discrepancy between previous findings by showing that the ALIX-NC interaction is only RNA-dependent when cellular membranes are disrupted by detergent treatment, supporting a model in which NC adopts a recognizable conformation either when it is bound to RNA or to membrane lipids [28]. This work has parallels with the finding that the MA domain also binds RNA but that this interaction is competitively disrupted by membrane lipids [64]. In combination these data suggest that RNA and lipids compete for binding at both the MA and NC domains of Gag.

HIV-1 is remarkably efficient at packaging RNA into virions, with a recent study finding that >90% of virions contain gRNA [92]. An attractive hypothesis would be that the binding of gRNA to NC acts as a switch to initiate the cascade of events that occur when the ESCRT complexes are recruited by Gag. It is well known that Gag expressed in isolation in mammalian cells is able to form immature virus-like particles that bud from the plasma membrane [93], however in the context of viral infection it would be advantageous for immature virions that have successfully encapsidated gRNA to be released preferentially to the release of empty virions.

## 8. Maturation of the Viral Core

During, and after, budding of virions from the plasma membrane, the Gag polyprotein undergoes a coordinated series of cleavages by the viral protease [94,95,96]. The relative timing of each cleavage step is critical for production of mature, infectious virions. There is strict coordination of Gag processing with virus budding, since delays in budding cause critical enzymes liberated by proteolytic cleavage to diffuse back into the cytoplasm to yield non-infectious virions [97]. Furthermore, disruption of any of the cleavage steps using mutagenesis or pharmacological agents produces non-viable virions with abnormal core morphologies [98,99,100]. The final step of cleavage, namely, removal of the spacer peptide SP1 (also termed p2) from CA, is particularly important as it triggers the dramatic structural rearrangement of the core from a spherical to a conical morphology [98].

Mutations in the RNA packaging signals in both HIV-1 and HIV-2 result in Gag processing defects that affect particle morphology and, thus, infectivity [22,23,24]; similarly, mutations in the NC domain of Gag influence Gag processing [25]. In HIV-2, the Gag processing defect appears to be tightly linked to the ability of the genome to dimerize [24]; however, the same dependency has not been observed in HIV-1. The viral protease is a homodimer derived from pairing of monomers from individual Gag-Pol molecules; thus, the processing cascade is initiated by juxtaposition of two Gag-Pol molecules to form a relatively unstable protease that is only capable of intramolecular cleavage [95]. An attractive model might be that the dimeric genome acts as a tether to bring the first Gag-Pol molecules together, however in the HIV-1 and HIV-2 studies the Gag processing defects appear to affect specific steps of cleavage, rather than its initiation, implying that the mutations in the RNA have stage-specific effects.

Mutations in and around the packaging signal region of the HIV-1 genome drive evolution of compensatory mutations in long-term culture [22,101,102,103]. Intriguingly, these are found in the Gag coding region and seem to act at the protein rather than at the RNA level [104]. They lead to restored packaging of gRNA and inhibition of packaging of spliced viral and cellular RNAs [104,105], and also restore processing of CA-SP1 cleavage [22,103,104], suggesting that gRNA binding to Gag and CA-SP1 cleavage are linked.

Although substituting the NC domain of Gag with a leucine zipper motif in the context of a full-length proviral clone does not completely block production of mature virions, the majority of particles display mildly or severely aberrant morphology [9]. This finding suggests that packaging of gRNA is important to ensure that the majority of particles adopt a mature, infectious form. It has also recently been demonstrated in vitro that the activity of the HIV-1 protease, itself, is markedly increased in the presence of RNA, although, curiously, the same effect is not observed for the HIV-2 protease [106].

## 9. Conclusions

Genome packaging in RNA viruses encompasses a multifaceted interplay between the viral genome and both cellular and viral factors. Constraints on genome size mean that RNA viruses have evolved many stratagems to enable them to use sequences, structures, and switches in gRNA to direct their assembly without requiring additional coding capacity. Increasing knowledge of the mechanisms used to coordinate this process will teach us more about basic molecular biology and provide new targets for treatment of RNA virus infections, particularly where the sequences involved are in highly-conserved regions of the genome, such as the 5’ UTR of HIV-1, which make the barriers to drug resistance high.
